# Ranging Behaviour of Commercial Free-Range Laying Hens

**DOI:** 10.3390/ani6050028

**Published:** 2016-04-26

**Authors:** Leonard Ikenna Chielo, Tom Pike, Jonathan Cooper

**Affiliations:** Animal Behaviour, Cognition and Welfare Research Group, University of Lincoln, Lincoln LN6 7TS, UK; tpike@lincoln.ac.uk (T.P.); jcooper@lincoln.ac.uk (J.C.)

**Keywords:** ranging behaviour, free-range laying hens, feather condition, enrichment, ecological survey

## Abstract

**Simple Summary:**

Commercial free-range production has become a significant sector of the fresh egg market due to legislation banning conventional cages and consumer preference for products perceived as welfare friendly, as access to outdoor range can lead to welfare benefits such as greater freedom of movement and enhanced behavioural opportunities. This study investigated dispersal patterns, feather condition and activity of laying hens in three distinct zones of the range area; the apron area near shed; enriched zone 10–50 m from shed; and outer range beyond 50 m, in six flocks of laying hens under commercial free-range conditions varying in size between 4000 and 24,000 hens. Each flock was visited for four days to record number of hens in each zone, their behaviour, feather condition and nearest neighbour distances (NND), as well as record temperature and relative humidity during the visit. Temperature and relative humidity varied across the study period in line with seasonal variations and influenced the use of range with fewer hens out of shed as temperature fell or relative humidity rose. On average, 12.5% of the hens were observed on the range and most of these hens were recorded in the apron zone as hen density decreased rapidly with increasing distance from the shed. Larger flocks appeared to have a lower proportion of hens on range. The hens used the range more in the early morning followed by a progressive decrease through to early afternoon. The NND was greatest in the outer range and decreased towards the shed. Feather condition was generally good and hens observed in the outer range had the best overall feather condition. Standing, pecking, walking and foraging were the most commonly recorded behaviours and of these, standing occurred most in the apron whereas walking and foraging behaviours were recorded most in the outer range. This study supported the findings of previous studies that reported few hens in the range and greater use of areas closer to the shed in free-range flocks. This study suggests that hens in the outer range engaged more in walking and foraging activities and showed signs of better welfare than those closer to the shed.

**Abstract:**

In this study, the range use and behaviour of laying hens in commercial free-range flocks was explored. Six flocks were each visited on four separate days and data collected from their outdoor area (divided into zones based on distance from shed and available resources). These were: apron (0–10 m from shed normally without cover or other enrichments); enriched belt (10–50 m from shed where resources such as manmade cover, saplings and dust baths were provided); and outer range (beyond 50 m from shed with no cover and mainly grass pasture). Data collection consisted of counting the number of hens in each zone and recording behaviour, feather condition and nearest neighbour distance (NND) of 20 birds per zone on each visit day. In addition, we used techniques derived from ecological surveys to establish four transects perpendicular to the shed, running through the apron, enriched belt and outer range. Number of hens in each 10 m × 10 m quadrat was recorded four times per day as was the temperature and relative humidity of the outer range. On average, 12.5% of hens were found outside. Of these, 5.4% were found in the apron; 4.3% in the enriched zone; and 2.8% were in the outer range. This pattern was supported by data from quadrats, where the density of hens sharply dropped with increasing distance from shed. Consequently, NND was greatest in the outer range, least in the apron and intermediate in the enriched belt. Hens sampled in outer range and enriched belts had better feather condition than those from the apron. Standing, ground pecking, walking and foraging were the most commonly recorded activities with standing and pecking most likely to occur in the apron, and walking and foraging more common in the outer range. Use of the outer range declined with lower temperatures and increasing relative humidity, though use of apron and enriched belt was not affected by variation in these measures. These data support previous findings that outer range areas tend to be under-utilized in commercial free-range flocks and suggest positive relationships between range use, feather condition and increased behavioural opportunities and decline in the use of range in cold and/or damp conditions.

## 1. Introduction

Free-range egg production has become popular due to consumer interest in welfare friendly products and the banning of conventional wire cages across the European Union (EU) in January 2012. As a consequence, free-range production approaches 50% of the fresh egg market in the UK [1]. EU Council directive [2] requires that stocking density must not exceed 2500 hens per hectare, which is equivalent to four square metres per bird. A number of quality assurance schemes include further requirements, for example The Royal Society for Prevention of Cruelty to Animals (RSPCA)’s Assured, British Egg Industry Council’s Lion Brand and Noble Food’s Happy Egg brand stipulate flock sizes of no more than 16,000 birds, and where flocks exceed 6000 hens, the flock should consist of colonies or sub-flocks of no more than 4000 birds. To meet these requirements, commercial free-range systems typically consist of large flocks of up to 16,000 birds, housed in a large permanently built shed in a large field (about six hectares for a 16,000 bird flock). 

Several studies have reported limited outdoor use in free-range laying hens [3,4,5,6,7] and this pattern in the use of range may be associated with a number of welfare problems, e.g., feather pecking, cannibalism and parasitic fouling of pasture in the extensively used areas [8,9,10]. The number of hens found outdoors has been reported to be inversely related to flock size [4,9,11,12,13] with a smaller fraction of the population using the range in larger flocks. Ranging patterns of hens have also been found to be influenced by strain differences [14], season and/or weather conditions [3,4,15], early outdoor rearing experience [3], age of flock [11,14], pop-hole availability [3,16], light intensity in the shed [3] and presence of keel bone fractures [15]. Overall, tree cover and artificial shelters have been utilized to attract hens into the range [3,5,17]. These resources are thought to also provide additional behavioural opportunities to the hens, though behaviour has tended not to be studied in detail, except for direct and indirect assessment of feather pecking.

Hens are thought to accrue a number of welfare benefits when they use the range [7,18]. Savory [19] reported a link between tree cover availability, use of range and injurious feather pecking. Increased use of the range has also been associated with lower prevalence of injurious feather pecking in free-range laying flocks in a number of studies [10,11,17,20,21,22]. Nicol *et al*. [17] reported a beneficial effect of increased use of range with hens showing a nine-fold reduction in feather pecking activities when more than 20% of hens used the range on sunny days, whilst Bright *et al*. [10] found that feather damage correlated negatively with percentage of canopy cover in end-of-lay hens. They suggested that providing 5% cover within 20–25 m distance from the laying hen house is beneficial to the improvement of feather condition and that injurious feather pecking was reduced when a higher proportion of hens use the range. 

This study further explored the ranging behaviour of free-range laying hens. Whilst previous studies have tended to focus on flock level measures of condition and use of outdoor areas, this study aimed to provide a more detailed assessment of dispersal and behavioural patterns. The outdoor area was divided into 3 zones based on proximity to shed and available resources. These were: apron (0–10 m from shed normally without cover or other enrichments); enriched belt (10–50 m from shed where resources such as cover, trees, bushes or saplings and dust-baths were provided); and outer range (beyond 50 m from shed with no cover and mainly grass pasture). The feather condition, NND and behaviour of the hens in different outdoor areas were sampled to determine the impact of location on these parameters. In addition, this study used line transects (a technique derived from ecological census) as a further means of measuring hen numbers and dispersal patterns in the three outdoor zones. We predicted that there would be a decline in the number of hens per unit area with distance from shed and that increasing flock size would reduce range use as found in previous work. This study aimed to provide evidence on how these two factors interacted and the differences in behaviour, dispersal patterns and feather condition between zones.

## 2. Materials and Methods

### 2.1. Animals and Management

This study was approved by the University of Lincoln’s College of Science Ethics Committee and was carried out using six flocks of commercial laying hens at four farms in Lincolnshire. All six flocks supplied the Happy Egg Company established by Noble Foods UK Ltd. (Oxford, UK). The study population consisted of medium hybrid lines commonly used in egg production: three flocks of Hyline and three flocks of Lohmann Brown hens, with population sizes ranging from 3900 to 23,548 birds and aged between 27 and 55 weeks of age. 

Continuous lighting was provided in the sheds for a minimum of eight hours each day and the hens had daytime access to the outdoor range at twenty weeks of age through the pop holes measuring 45 cm × 2 m, located on the two sides of the sheds. There was at least one pop hole per six hundred hens usually opened at 9:00 a.m. each morning and closed at dusk (4:00 p.m. to 6:00 p.m. during the study). The hens were provided with range enrichments and trees to comply with specification of Happy Egg Company. In addition to complying with the requirements of the EU Council Directive [2], the hens in this system had access to additional resources (activity kits) outside the shed and were the same on each farm, comprising of one set of mini shelter, dust bath and a perch per 4000 hens in the outdoor area. Trees were planted between the distance of 10 m and 50 m from the sheds, with the majority of manmade structures located 15–35 m away from the shed. These resources were thought to encourage hens to utilize the outdoor area by providing shelter and increasing behavioural opportunities, though at the time of study the trees were saplings, so canopy cover was limited. 

The study was completed between November 2011 and February 2012. Each flock was visited on four different occasions (*i.e.*, each flock was visited four times before another flock was visited) for data collection giving a total of twenty-four sample visits for the study. At least a 48-hour gap was allowed between farm visits to comply with Noble Food’s bio-security requirements.

### 2.2. Sampling Areas

On first arrival, each flock was surveyed for key environmental features including location of shed within field, field boundaries, location and number of pop-holes and the distribution of outdoor resources. Bamboo poles (1 m in height) were then used to divide the outdoor area into zones and to produce 4 lines of transects running perpendicular to the shed. Pilot studies for previous studies [23] had indicated that whilst these poles provide a short term point of interest to hens when initially placed in the ground, the hens rapidly habituated to their presence and the poles had no influence on hens’ location after half an hour. Poles were placed in pairs, every 10 m from the shed for 110 m to indicate the quadrats. This had the effect of firstly providing sighting lines parallel to the shed to allow the distance from the shed to be estimated. They also produced the line transect from the edge of shed to 110 m that was made up of eleven 10 m × 10 m quadrats. Where sheds were located centrally in fields and had over 100 m on either side, then two transects were arranged on either side of the shed. Where the sheds were on the edge of the field, with all or most useable area on one side, all four transects were arranged on that side. 

Outdoor areas of commercial free-range systems often have features that can be used to differentiate discrete zones located at specific distances from the shed and with different enrichment resources. In this study we defined three discrete zones whose features were common across all the six flocks. These were the apron, enriched and outer range zones. The apron zone was defined as the area between 0 m to 10 m from the shed. There were no additional enrichment resources in this area and no tree cover. Ground vegetation was sparse, with soil, slats, concrete or pebbles covering most of the area. This constituted on average 4.1% of the available outdoor area. The enriched belt covered the area between 10–50 m from the shed and manmade enrichments were located in this area with natural cover in the form of plantations of tree saplings. Ground vegetation varied from low grass pasture, with patches of taller perennial plants such as nettle and patches of bare earth, particularly where the hens had formed scrapes or dust baths. This constituted on the average 21.2% of the available outdoor area. The outer range was defined as the outdoor area 50 m and beyond from the shed and this was the largest part of the outdoor area spanning from the end of the enriched zone to the field boundaries and covered on average 74.7% of available outdoor area. This area mainly consisted of grass pasture, which tended to have low sward during the study period. The boundary for all flocks was 2 m tall electrified wire fence to prevent hens leaving field and deter ground predators from entering the enclosure. 

### 2.3. Data Collection

A general head count was conducted around 12:00 p.m. to determine the total number of hens outdoors in each flock. This involved a brisk walk of the flocks to count the number of hens in apron, enriched and outer range zones, which were combined to provide the estimate for the entire flock. These were used to calculate the percentage of the flock in each area based on farm records of flock size at time of survey. Furthermore, the population density could be calculated in terms of hens per square metre and range area per hen in apron, enriched belt and outer range.

Hen numbers and distribution across zones was also recorded using ecological census techniques utilized by Cooper and Hodges [23]. This was conducted by counting the hens in each quadrat of each transect four times during each visit. Head counts were carried out at 10:00 a.m., 11:00 a.m., 1:00 p.m. and 2:00 p.m. and during the counts, the potential observer influence associated with head counts was minimized by maintaining a distance of 15 meters from the hens. In addition to heads counts, twenty hens were sampled in each zone for NND, feather condition and behaviour. Where there were less than twenty hens in a quadrat, all the hens were sampled. The hen closest to the observer and every second hen was sampled and their immediate activity was categorized using an ethogram of 17 mutually exclusive behaviours ([24], Table 1). 

A visual assessment of plumage condition of four different body parts (neck, chest, back and sides) was carried out using a six point scoring scale [25]. In this method, values from 0 (best feathers) to 5 (worst feathers) were assigned to each body parts (see Table 2 for scale values and descriptions) with respect to the degree of damage or no damage to the feathers. The feather condition of the hens was effectively scored from a distance of 5 m to minimize disturbance to the focal animal and flock in general. Bright *et al*. [26] reported a strong positive correlation between feather condition scores recorded from distance and scores from close inspection following capture, which suggests that distance feather scoring techniques are reliable and practical on a commercial scale. Feather scoring and behavioural observations were carried out after the head counts.

NND was measured using 1 m poles to mark the locations of the focal bird and its nearest neighbour and the distance measured by running a portable 25 m tape between the poles. The approximate location of chickens and nearest neighbour was noted and measured using measuring tape with the help of a field assistant. This approach was effective at estimating distances to nearest 0.2 m over distances of up to 2 m, but accuracy declined above this distance so distances above 2 m where estimated to the nearest 1 m. 

### 2.4. Weather Measurements

The temperature and relative humidity of sites were measured using a simple indoor/outdoor thermo-hygrometer. Upon arrival, the thermo-hygrometer was positioned in the open outdoor area mid-way between shed and end of range at 2 m above the ground level. The temperature and relative humidity was recorded 4 times per day on each visit at 10:00 a.m., 11:00 a.m., 1:00 p.m. and 2:00 p.m.

### 2.5. Statistical Analysis

All the data collected was analysed using IBM SPSS 20.0 statistical software (IBM Corporation, Armonk, NY, USA). Temperature and relative humidity data met requirements of parametric statistics, so the effects of time of day were assessed using general linear model analysis of variance (GLM ANOVA) with time of day as fixed factor and flock as a random factor. The average measure of temperature and relative humidity for each visit day was then used to investigate the effect of these measures on total number of hens out of shed. We had planned to investigate the effects of flock size, age and strain as factors in the analysis, however, as it was not possible to balance these between flocks (See Table 3), we instead treated flock identity as a random factor across all analysis.

GLM analysis was performed on the quadrat distribution data to determine if the fixed factors (zone and time of the day) had influence on the distribution of the hens. Flock identity was treated as a random factor whereas age, strain, flock size, temperature, and relative humidity were fitted as covariates. As there were a number of potential explanatory variables used in the analysis, a step-wise model simplification process was carried out. The residual of the model used in the analysis was found to be normally distributed using a histogram and therefore no data transformation was required before analysis. 

NND data was also analysed using GLM approach. A separate model was developed using a similar step-wise simplification procedure as the hen distribution to determine if the location of hens influenced their distances away from the nearest hens. In this model, zone was fitted as a fixed factor and flock was treated as a random factor. 

Feather scores from four key body parts (head, neck, chest and back) were explored for descriptive statistics and a GLM analysis was carried out to determine if there was a relationship between feather condition of the hens and the outdoor zone they were found. A fitted feather score model was achieved using a similar approach as in the above analysis.

Behavioural data were explored for descriptive statistics to determine the relative abundance of each of the behaviours recorded. The results of the descriptive analysis revealed that some of the behaviours were not recorded at all or rare and for this reason, only the major behaviours were analysed further using GLM. A step-wise model simplification process was also carried out as in the models above to determine the influence of the zones on the behaviours. 

The means and standard error of means for the estimates of distribution, feather scores, NND, behavioural occurrence, temperature and relative humidity are presented in the result section of this article. Further *post hoc* tests were carried out on all the significant variables and interactions in each model using Bonferroni correction factor to determine the pairs that were significantly different from each other.

## 3. Results

### 3.1. Weather

Average temperature sampled on visits was 8.56 ± 0.42 °C (range from 0.00 °C to 17.5 °C), and the relative humidity was on average 72.6 ± 1.5% (range from 42.0% to 99.0%). The temperature and relatively humidity varied across the study period, broadly in line with seasonal variations and consequently there was significant variation in both temperature (F_5_ = 4.95, *p* < 0.001) and relative humidity (F_5_ = 3.38, *p* = 0.008) between flocks. There was no effect of time of day on either temperature or relative humidity.

### 3.2. Number of Hens Outdoors and Their Distribution

The mean number of hens out of shed was 1142 ± 91 which represented an average 12.5% of the flock. An average head count of 530 ± 37 was found for the apron area near the shed (5.4% of flock), whereas 401 ± 51 hens were recorded in the enriched belt (4.3%), and 211 ± 44 hens (or 2.8% of flocks) were recorded in the outer range. As the majority of the outdoor area was the outer range, with apron and enriched belts only covering about a quarter of available outdoor area, these resulted in considerable variation in stocking density between the three areas. There were on average 0.31 hens/m^2^ on the apron (equivalent to 3.23 m^2^ per hen), compared with 0.048 hens/m^2^ in the enriched belt (or 20.8 m^2^ per hen) and 0.0086 hens/m^2^ of outer range giving on average 116 m^2^ per hen. There was a significant difference between flocks in percentage of hens on the range (F_5_ = 20.1, *p* < 0.001), which varied from 35.1% in the smallest flock to 3.0% in the largest flock (Table 3). 

### 3.3. Number of Hens in the Quadrats

**Figure 1:**
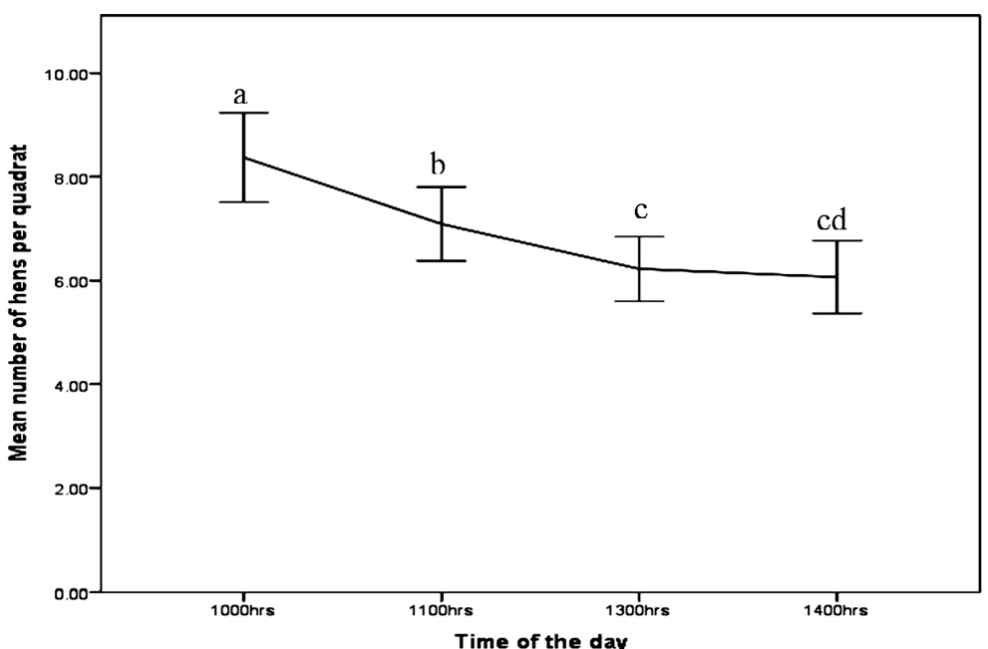
The means of number of hens (±SE) recorded at different times of the day. ^a,b,c,d^ Means of times with different superscripts are significantly different (*p* < 0.05).

The distribution of hens across quadrats was influenced by the time of day (F_3_ = 63.97, *p* < 0.05) with the greatest number of hens outside at 10:00 a.m. (Figure 1). Outdoor use decreased significantly through the day but showed similar number of hens between 1:00 p.m. and 2:00 p.m. There were more hens in the apron area least in the outer range and intermediate in the enriched zone at all the time periods (Table 4: F_6_ = 30.59, *p* < 0.05). 

**Figure 2:**
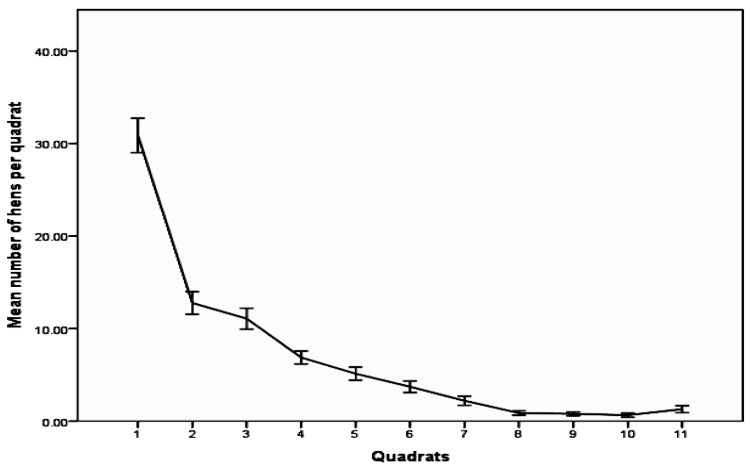
The mean (±SE) number of hens per 100 m^2^ quadrat from first quadrat on apron, through enriched belt (2–5) and outer range (6–11) showing sharp decline in numbers with distance from shed.

The counts of hens in the quadrats that made up the line transects were consistent with the findings of the overall head counts, with numbers falling with distance from shed (F_2_ = 352, *p *< 0.001; Figure 2). Quadrat 1, which included the apron, had an average of 30.87 ± 0.44 hens, quadrats 2 to 5 in the enriched belt had on average 8.97 ± 0.22 hens, whilst quadrats 6–11 in the outer range had a mean of 1.60 ± 0.18 hens per 100 m^2^ quadrant. These counts equated to about 3.24 m^2^ per hen in the apron quadrats, 11.1 m^2^ per hen in the quadrats from the enriched belt and 62.6 m^2^ per hen in the outer range quadrats. There was also an effect of flock (F_5_ = 29.1, *p* < 0.001), with smaller flocks having higher numbers of hens per quadrat than larger flocks (Table 3).

There was an effect of temperature on the number of hens per quadrat (F_1_ = 3.02, *p* < 0.001) with more hens as temperature rose. There was however, no overall effect of relative humidity (F_1_ = 3.02, *p* > 0.05) on outdoor use.

### 3.4. Nearest Neighbour Distance

The NND of hens outdoors and was found to differ between the zones (F_2_ = 435, *p* < 0.001). NND was found to be greatest in the outer range (5.67 ± 0.15), least in the apron (1.62 ± 0.05) and intermediate in the enriched belt (2.40 ± 0.08) so distance between hens increased with increasing distance from the shed (Table 5). There was no effect of flock on the NND (F_5_ = 2.69, *p* > 0.05).

### 3.5. Feather Condition of the Hens

The results showed that feather loss was worst in the neck area (0.609 ± 0.013), followed by chest (0.192 ± 0.008) and back (0.078 ± 0.005), with the side having the best condition scores (0.003 ± 0.001) (F_3_ = 1461, *p* < 0.001). *Post hoc* tests revealed that the hens in the range zone had the best overall feather condition whereas apron had poorer feather condition (see Table 5). Feather condition also differed between flocks (F_5_ = 12.9, *p* < 0.001; Table 3) with the smallest flock having the best feather condition, followed by second smallest flock and no difference between the four larger flocks.

### 3.6. Behaviour of the Hens

Standing (24.8% of samples), pecking (19.8%), walking (26.6%) and foraging (20.6%) were the most recorded behaviours, representing over 90% of the overall activity of hens that were sampled. Of the remaining behaviours only preening (3.4%), sitting (2.6%) and ground scratching were found in more than 1% of samples. Running (0.4%), dust-bathing (0.2%), perching (0.1%) and shaking (0.1% of samples) were rarely sampled. The remaining activities in the ethogram including aggression and comfort behaviours such as wing flapping were not recorded.

To provide a measure of the effect of location on the relative abundance of each activity we compared the incidence of the most common activities in each zone. The results of the relative occurrence of these behaviours in different zones are presented in Table 6. The results showed that standing behaviour differed between the three zones (F_2_ = 52.9, *p *< 0.001) and was recorded most in the apron zone. Pecking behaviour was less common in the outer range than in the other zones (F_2_ = 24.3, *p* < 0.001). Walking (F_2_ = 14.4, *p* < 0.001) and foraging (F_2_ = 53.6, *p* < 0.001) activities occurred most in the outer range zone with mean difference between the zones being significant. There was no effect of flock on behavioural measures.

## 4. Discussion 

The findings of the present study were broadly in line with other research in commercial free-range layer systems regarding range use, in particular that population density declines with distance from shed (e.g., [5,16,17]) and that a lower proportion of the flock are found outdoors as flock size increased (e.g., [4,12,13]). This study also found evidence for a positive relationship between good range use and feather condition (e.g., [17,21,22]) and reduced general range use in colder (e.g., [3,4,15]). The novel sampling methods and additional measures used in this study provided more detailed data on the use of range as well as the relationship between range use, behaviour and feather condition.

The total head count showed that 12.5% of the hens in the sampled flocks were found outdoors at noon. In this study, we did not directly assess the effect of flock size in our analysis due to confounding effects of other flock variables such as age and strain. Nevertheless the variation in both total percentage of the hens counted out of shed and the number of hens counted using quadrat approach suggests that as flock size increased, the use of ranging areas decreased and this is consistent with the findings of a number of other studies [4,9,11,12,13]. It may have been more demanding for hens in larger flocks to come out of the shed because of the number of hens they will encounter before accessing the outdoor area. In addition, hens in larger flocks require bigger sheds and this will mean that they will walk longer distance to access the range. 

Most of the hens found outdoors were recorded in the apron zone and the preference for a closer proximity could be an anti-predator strategy as suggested by Nagle and Glatz [5]. They reported more hens in the range (including the farther areas) when shelterbelts were provided, and suggested these provided shelter from predation [6,27] though shelter from wind, rain or direct sunshine would also be a benefit. Hegelund *et al*. [4] also observed that artificial cover attracted more hens into the range and away from the immediate strips of the shed. The work of Zeltner and Hirt [28] supported the findings of this study and showed that hens were less likely to use the range when no overhead cover was provided. The trees planted in the outdoor range of the flocks used for this study were mainly saplings, which did not provide high levels of cover for the hens. Previous work [23] suggests that although saplings can increase range use, full or nearly full canopy cover has greatest impact on the range use.

It is worth noting that in this study, there were differences between the hen densities derived from head counts and those from the quadrats for the outer range and enriched belt, but not the apron. The head counts of hens out of shed gave an estimate of 0.31 hens/m^2^ of which is equivalent to 31.0 hens per 100 m^2^ of apron, and similar to the average of 30.9 calculated from quadrat counts. In contrast, twice as many hens were recorded in the enriched belt (9.0 compared with 4.8 based on data from entire enriched belt) and outer range (1.6 hens compared with 0.9 across entire outer range) using the quadrat method compared to general head counts. This suggests that the area covered by the line transects in the enriched belt and outer range were more attractive than other areas of enriched belt and outer range zones. This may simply be a matter of ease of access, in that transects were in line with the sides of sheds where most pop holes would be located or because of the movement of the observer along the transect lines during observations which has the potential to attract more hens to this area compared to the less frequently walked areas of the range. 

Alternatively, the higher hen density recorded in the quadrat counts may be related to the specific enrichments provided in these locations e.g., the young tree plantations found across the entire enriched belt, the sampled areas were also the locations of the activity kits provided as part of the Happy Egg Companies flock requirements. These normally consisted of covered dust baths, perch and mini-shelters with one set of kits per 4000 hens, and may have provided additional shelter or behavioural opportunities to attract birds compared to young trees alone. A more detailed study of the impact of additional manmade enrichments and their locations on hen movement would be able to differentiate between these two explanations.

The hens used the range most in the morning but the number of hens outdoors dropped as the day progressed. The interaction between zone and time of the day showed that the hens used the apron most at all times. This study supports other findings [4,5,15] where free-range hens were reported to use the outdoor run most in the morning and evening. In the present study, the hens were observed between 10:00 a.m. to 2:00 p.m. and because of this, the use of range beyond the time was not assessed. The differences in atmospheric conditions across the day may explain why the hens prefer to range more in the morning (e.g., Nicol *et al*. [17]), however in this study, although temperature did affect numbers hens sampled, these factors did not consistently vary with time across the sampling days. Alternatively, the higher numbers at 10:00 a.m. may reflect high numbers of hens using range resources soon after pop-hole opening at 9:00 a.m. 

There was a relationship with general distribution of hens and of the two weather variables measured; higher temperature had largely positive effect on the numbers counted in quadrats, whereas negative impact of relative humidity that would be predicted from other studies was found in this study. Hegelund *et al*. [4] reported a parabolic relationship between temperature and number of hens in the range and they showed that range use increased up to a maximum temperature of 17 °C then a corresponding decrease in range use at temperatures greater than 17 °C. The maximum recorded temperature for this study was 17.5 °C and appeared to encourage more hens to range. Gilani *et al*. [3] reported a similar range use pattern and evidence from their work found that more hens ranged away from the sheds when the relative humidity level was low, on cooler days and with low rainfall. Hegelund *et al*. [4] also reported that wet and rainy conditions had negative influence on the use of range. These findings point to the potential importance of shelter to protect from adverse weather.

The results suggested that location of the hens affected their behaviour. The most commonly recorded activities were standing, pecking, walking and foraging. Comparison of the number of hens engaged in each of these behaviour indicated that standing and pecking were most often observed in the hens found in the apron area, and that walking and foraging were commonly observed in the outer range. The latter may be a reflection of the availability of the large, open space of the outer range and the distance hens would travel to search for resources in the outer range. In contrast the apron provided relatively little additional environmental resources but was close to the indoor environment so required little movement. It is also noteworthy that no aggressive behaviour was recorded in any of the hens sampled, suggesting this behaviour was rare outside of the shed environment. This study did not sample behaviour inside the shed, which may have provided greater potential to detect incidences of feather pecking and aggression due to higher stocking density and different resource availability. 

The results indicated that the NND increased as you go away from the shed. The hens showed the least NND in the apron, greatest in the outer range zone and intermediate in the enriched belt. Based on the results of the total and quadrat head counts, apron zone had the greatest number of hens and covered less than a quarter of the available outdoor area, thereby making it the most densely populated outdoor zone. As the number of hens dropped from the shed, there was more usable space available to individual hens (decreased stocking density), which in turn resulted in the increasing NND recorded in this study. The hens in the outer range may have moved to this area to avoid aggression or competition for the resources in the zones near the shed and may have preferred to maintain greater distances from other hens to avoid such aversive experiences. 

The results of feather score analysis showed that the sides had the most intact feathers and neck had the most damaged feathers. There was an effect of flock on feather condition with the two smallest flocks having significantly better feather scores than the four larger flocks. Before drawing too strong a conclusion from this, it is worth noting that only six flocks were included in this study, that they represented a relatively restricted age range (five flocks between 48 and 55 weeks of age and one flock of 27 weeks), and that the feather condition of the hens was generally very good. Normally feather condition would be expected to decline with age, and a larger number of flocks covering a greater age range would be expected to find a significant age effect. Nevertheless, the better feather condition associated with smaller flocks in this study that made more use of the outside environment is noteworthy. 

Hens in the outer range zone had the best feathers (in all body parts) whereas apron recorded the worst feathers. The feather condition of hens in the enriched zone was better than those found in the apron but worse than their counterparts in the outer range zone. Although we found some variation in plumage, overall feather condition was excellent and many hens in outer range, enriched and apron zones showed no evidence of feather damage. Bilcik and Keeling [25] suggested that ease of feather removal and the ease of access to different parts of the body may have resulted in specific feathers being attractive targets for pecking in laying hens; they have been reported to prefer shorter semi-plumes than longer ones [29]. Wing feathers have stronger shaft and are longer than the neck feathers, which may have resulted in less damages recorded in the wing feathers. High concentration of birds and greater pecking activities in the apron may have resulted in the poor feather quality of hens in this area. Range zone offered more foraging opportunities and hens in this zone foraged more and had better chances of avoiding competitive locations. Huber-Eicher and Wechsler [30] reported an inverse relationship between feather pecking and foraging behaviour in laying hens and in the present study, more hens in the range zone performed foraging behaviour at greater NND, which may have resulted in less feather damage recorded for the hens found in this area. 

## 5. Conclusions

These data supports previous studies that reported few hens in the outer range of free-range flocks. Hens that range further from shed were, however, more likely to be engaged in walking and foraging, compared to more sedate birds in the apron and enriched area, and generally had better feather condition. These findings suggest hens that make use of outer range have better welfare than those that remain close to the shed and the direction of any causal relationships warrants further investigation. For example, better feathered hens may be more likely to use range because they find it less aversive than other hens, or their feather condition may be improved because of spending more time out on range. Similarly, hens may travel to outer range because they are actively seeking opportunities to engage in activities such as foraging, or they may tend to naturally disperse further from sheds, and exhibit more foraging in response to the cues they encounter once they reach the outer range.

## Figures and Tables

**Table 1:** Ethogram of behaviours under observation (Adapted from Buijs, [24]).

Activity	Description
Standing	Not moving on two feet, body not touching the floor
Sitting	Body and both hocks touching the floor underneath or directly on either side of the bird
Lying	Lying on its side, with both feet on the same side of the bird
Walking	Slow locomotion, the first foot is put down on the floor before the second one is lifted (without pecking or scratching)
Pecking	Pecking on the ground or objects
Foraging	Walking with pecking and scratching
Running	Rapid locomotion, the second foot is lifted before the first is set down
Preening	Moving the beak over the feathers
Feeding	Pecking at the feed in the feeder, or between such pecks
Drinking	Pecking at the drinker, followed by tilting of the head
Shaking	Rapid whole body movement mostly associated with ruffling of the feathers or shaking dust from the plumage
Aggression	Fights including pecking at another chicken
Dust bathing	Foot scratching and bill-raking the litter or lose soil, followed by vertical wing shaking, head rubbing, bill-raking and/or scratching with one leg whilst lying
Stretching	Elongation of the leg not associated with walking
Comfort behaviour	Includes wing flapping, body shaking, feather ruffling and tail wagging but not preening
Head flick	Rapid head movements in horizontal plane
Scratching	Stepping backwards whilst raking the feet across the floor

**Table 2:** Description of feather scoring method used to evaluate feather condition of the hens (adapted from Bilcik and Keeling, [25]).

Score	Body Feathers	Flight Feathers
0	Intact feathers	Intact feathers
1	Some feathers scruffy up to 3 missing feathers	Few feathers separated but not broken or missing
2	More damaged feathers, greater than 3 feathers missing	A lot of feathers separated and/or a few broken or missing
3	Bald patch <5 cm diameter or <50% of area.	All feather separated, a lot of broken or missing feathers
4	Bald patch >5 cm diameter or greater than 50% of area	Most of feathers missing or broken
5	Completely denuded area	Almost all feathers missing

**Table 3:** Flocks and their characteristics including strain, number of hens at time of study and age in weeks. Means for % of flock out of shed, hens per quadrat and feather score are reported for each flock.

Farm	Strain of Hens	Size at Time of Study	Age (Weeks)	% of Flock Out of Shed	Hens per Quad	Feather Score
1	Hyline	3900	55	35.1 ± 3.8 **^a^**	9.8 ± 0.5 **^a^**	0.14 ± 0.01 **^a^**
2	Hyline	7300	48	20.1 ± 2.4 **^b^**	8.1 ± 0.5 **^b^**	0.21 ± 0.01 **^b^**
2	Hyline	15,573	27	6.3 ± 0.8 **^c^**	5.3 ± 0.4 **^d^**	0.24 ± 0.01 **^c^**
3	Lohmann Brown	15,470	49	4.6 ± 1.1 **^c,d^**	6.6 ± 0.4 **^c^**	0.24 ± 0.01 **^c^**
4	Lohmann Brown	15,797	51	8.8 ± 0.7 **^c^**	6.7 ± 0.4 **^c^**	0.25 ± 0.01 **^c^**
4 *	Lohmann Brown	23,548	52	3.0 ± 0.5 **^d^**	5.1 ± 0.4 **^d^**	0.23 ± 0.01 **^c^**

**^a,b,c,d^** Means within a column with different superscripts differ significantly (*p* < 0.05); * Single shed housing two flocks of approximately 12,000 birds each.

**Table 4:** Mean numbers of hens in the apron, enriched belt and outer range areas for all the time periods.

Zones					
Time of the Day	Apron	Enriched	Range	F-Value	SEM
10:00 a.m.	41.49 **^a^**	9.97 **^b^**	1.79 **^c^**	834.76	0.36
11:00 a.m.	29.98 **^a^**	9.31 **^b^**	1.79 **^c^**	460.67	0.35
1:00 p.m.	24.35 **^a^**	8.27 **^b^**	1.83 **^c^**	340.77	0.33
2:00 p.m.	27.67 **^a^**	8.31 **^b^**	0.97 **^c^**	402.67	0.35

**^a,b,c^** Means within rows with different superscripts are significantly different (*p* < 0.05).

**Table 5:** NND and feather condition for hens in apron, enriched belt and outer range.

Zones					
NND	Apron	Enriched	Range	F-Value	SEM
	1.62 ^a^	2.40 ^b^	5.67 ^c^	435	0.14
Feather condition scores					
Neck	1.064 **^a^**	0.610 **^b^**	0.154 **^c^**	538.38	0.028
Chest	0.296 **^a^**	0.211 **^b^**	0.070 **^c^**	78.27	0.018
Side	0.006 **^a^**	0.002 **^a,b^**	0.000 **^b^**	3.52	0.012
Back	0.213 **^a^**	0.014 **^b^**	0.007 **^b^**	192.83	0.002
Mean feather scores	0.395	0.214	0.058		

**^a,b,c^** Means within rows with different superscripts are significantly different (*p* < 0.05).

**Table 6:** The mean occurrence of the four most recorded behaviours (from each daily sample of 20).

Behavioural States	Apron	Enriched	Range	F-Value	SEM
Standing	16.6 **^a^**	8.9 **^b^**	4.2 **^c^**	52.87	0.61
Pecking	9.5 **^a^**	10.9 **^a^**	3.4 **^b^**	24.27	0.57
Walking	9.2 **^a^**	8.3 **^a^**	13.2 **^b^**	14.40	0.49
Foraging	1.7 **^a^**	7.1 **^b^**	15.8 **^c^**	53.63	0.68

**^a,b,c^** Means within rows with different superscripts are significantly different.
